# Supplementary material for: Disruption of Bacterial Thiol-Dependent Redox Homeostasis by Magnolol and Honokiol as an Antibacterial Strategy
Source: Antioxidants (Basel). 2023 May 30;12(6):1180. doi: 10.3390/antiox12061180 (PMC10294794; doi:10.3390/antiox12061180)
Supplement: Supplementary file 1 [file antioxidants-12-01180-s001.zip › Supplementary Figure S1.pptx]

## Slide 1
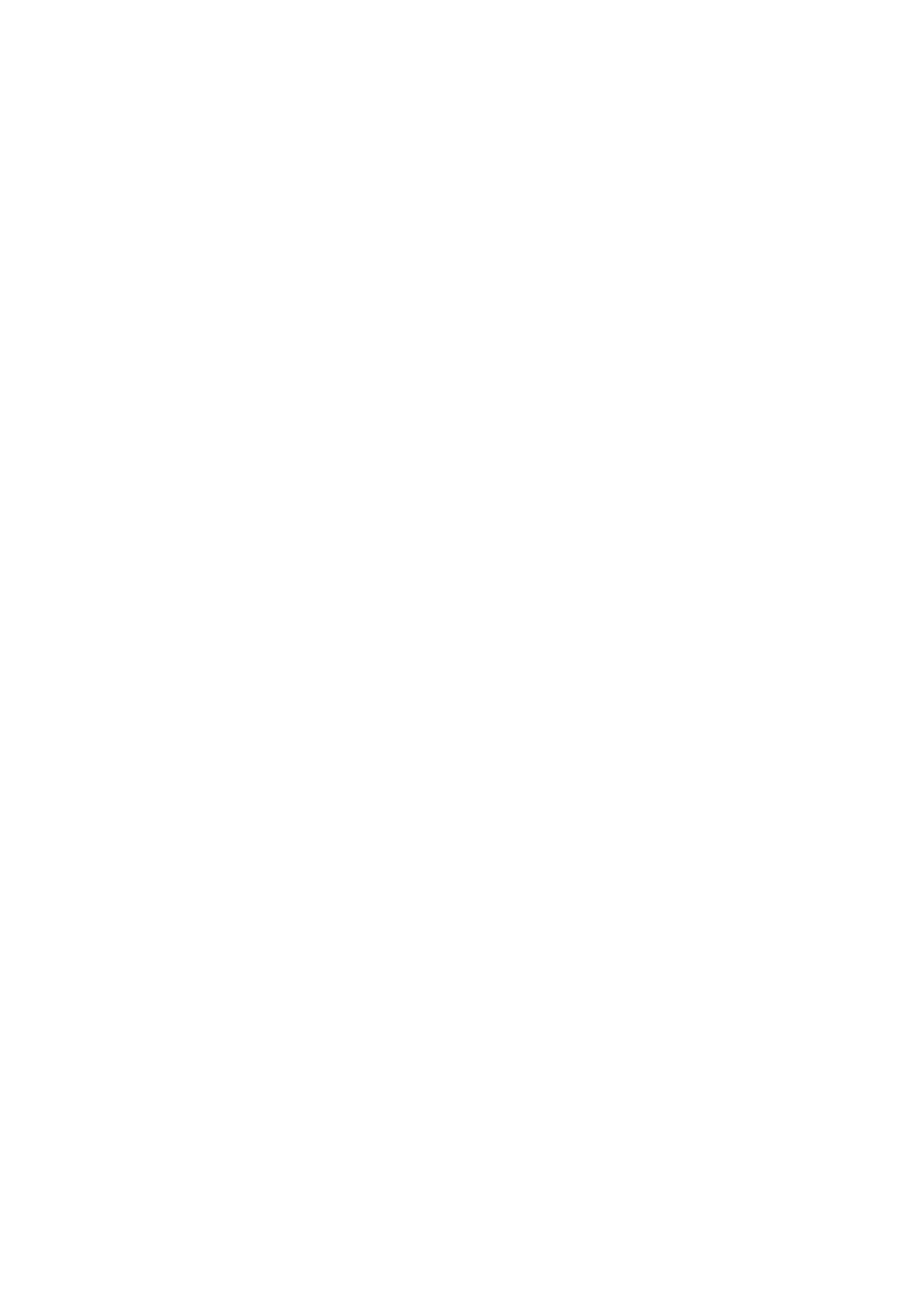

## Slide 2
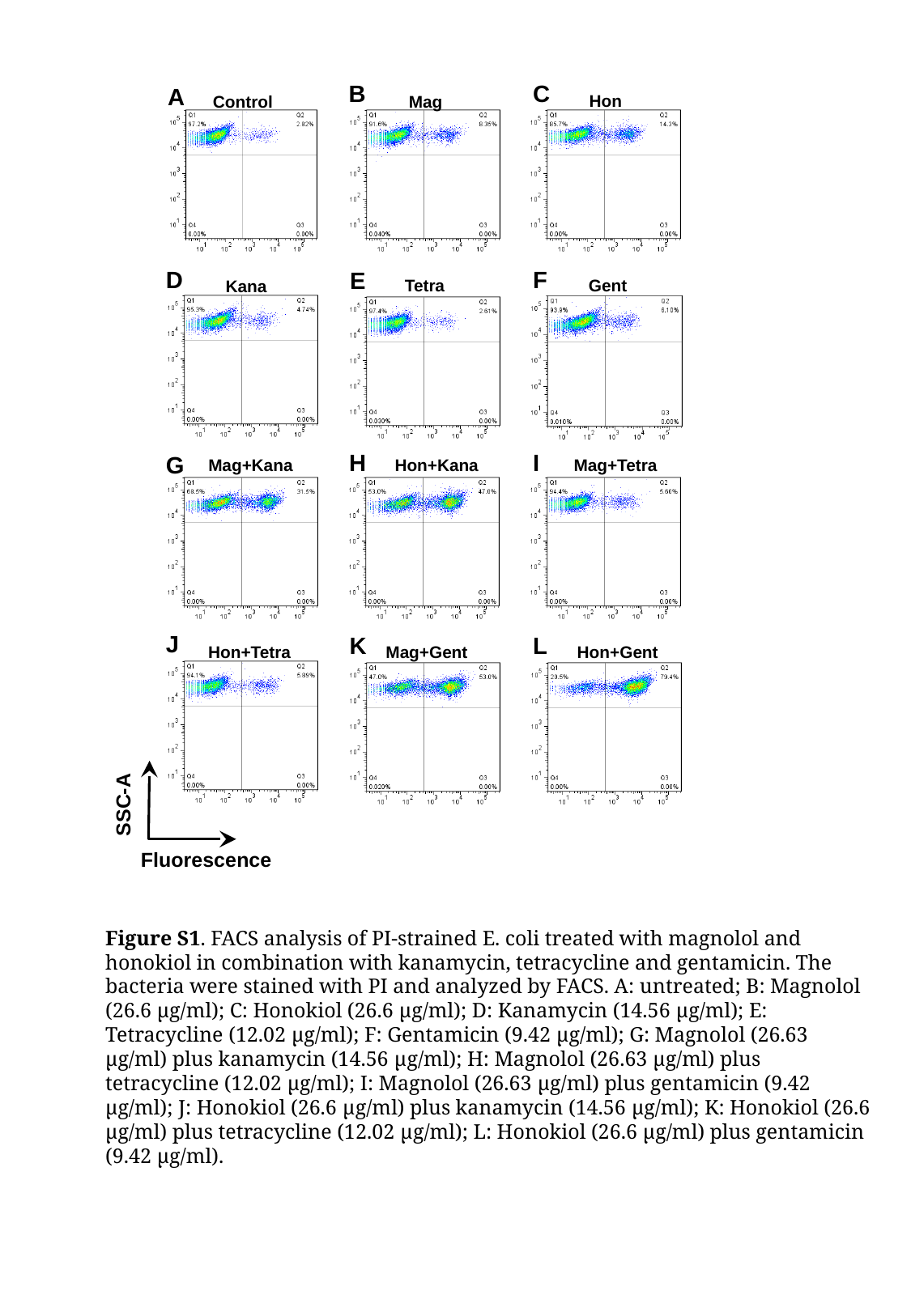

B
C
A
Hon
Mag
Control
F
D
E
Gent
Tetra
Kana
H
I
G
Hon+Kana
Mag+Kana
Mag+Tetra
J
K
L
Hon+Tetra
Mag+Gent
Hon+Gent
SSC-A
Fluorescence
Figure S1. FACS analysis of PI-strained E. coli treated with magnolol and honokiol in combination with kanamycin, tetracycline and gentamicin. The bacteria were stained with PI and analyzed by FACS. A: untreated; B: Magnolol (26.6 μg/ml); C: Honokiol (26.6 μg/ml); D: Kanamycin (14.56 μg/ml); E: Tetracycline (12.02 μg/ml); F: Gentamicin (9.42 μg/ml); G: Magnolol (26.63 μg/ml) plus kanamycin (14.56 μg/ml); H: Magnolol (26.63 μg/ml) plus tetracycline (12.02 μg/ml); I: Magnolol (26.63 μg/ml) plus gentamicin (9.42 μg/ml); J: Honokiol (26.6 μg/ml) plus kanamycin (14.56 μg/ml); K: Honokiol (26.6 μg/ml) plus tetracycline (12.02 μg/ml); L: Honokiol (26.6 μg/ml) plus gentamicin (9.42 μg/ml).
