# Supplementary material for: Disruption of Bacterial Thiol-Dependent Redox Homeostasis by Magnolol and Honokiol as an Antibacterial Strategy
Source: Antioxidants (Basel). 2023 May 30;12(6):1180. doi: 10.3390/antiox12061180 (PMC10294794; doi:10.3390/antiox12061180)
Supplement: Supplementary file 1 [file antioxidants-12-01180-s001.zip › supplementation 20230422.pdf]

Supplementary Table S1. MIC value of traditional Chinese medicines against various bacteria

MIC (mg/ml)	*E. coli* WT	*E. coli ΔoxyR*	*S. aureus*	*S. epidermidis*	*B. subtilis*	*B. cereus*
Ginger Magnoliae Officinalis Cortex	-	0.78	0.78	0.78	0.78	0.78
Cirsii Herba	-	1.56	-	-	-	-
Trberculate Speranskia Herb	-	6.25	-	-	-	-
Violae Herba	-	3.13	-	-	-	-
Genkwa flos	-	3.13	-	-	-	-
Ligustri Lucidi Fructus	-	3.13	-	-	-	-
Cynanchi Atrati Radix Et Rhizoma	-	3.13	-	-	-	-
Sinomenii Caulis	-	3.13	12.50	12.50	12.50	12.50
Plantaginis Herba	-	0.78	-	-	-	-
Lonicerae Japonicae Caulis	-	6.25	-	-	-	-
Leonuri Fructus	-	6.25	-	-	-	-
Drynariae Rhizoma	-	6.25	-	-	-	-
Zingiberis Rhizoma Praeparatum	-	3.13	-	-	-	-
Taraxaci Herba	-	1.56	-	-	-	-
Scutellariae Barbatae Herba	-	0.78	1.56	1.56	3.13	3.13
Viticis Fructus	-	3.13	-	-	-	-
Piperis Kadsurae Caulis	-	3.13	-	-	-	-
Faeces Trogopterpri	-	12.50	-	-	-	-
Sennae Folium	-	6.25	-	-	-	-
Dendrobii Caulis	-	6.25	-	-	-	-

Clematidis Radix Et Rhizoma	-	6.25	-	-	-	-
Epimedii Folium	-	3.13	-	-	-	-
Hedyotis Herba	-	6.25	-	-	-	-
Glycyrrhizae Radix Et Rhizoma	-	3.13	3.13	3.13	3.13	3.13
Thlaspis Herba	-	3.125	12.50	12.50	12.50	12.50
Chrysanthemi Flos	-	12.50	-	-	-	-
Mori Folium	-	3.13	-	-	-	-
Lotus Flower	-	1.56	12.50	12.50	12.50	12.50
Carthami Flos	-	1.56	6.25	6.25	6.25	6.25
Magnoliae Flos	-	6.25	-	-	-	-
Cacumen Platycladi	-	1.56	-	-	-	-
Cirsii Japonici Herba	-	3.13	-	-	-	-
Girald Acanthopanax Cortex	-	3.13	-	-	-	-
Menthae Haplocalycis Herba	-	0.78	12.50	12.50	12.50	12.50
Siegesbeckiae Herba	-	1.56	-	-	-	-
Prunellae Spica	-	0.78	3.13	3.13	3.13	3.13
Inulae Herba	-	1.56	12.50	12.50	12.50	12.50
Mori Cortex	-	-	1.56	1.56	3.13	3.13
Schizonepetae Herba Carbonisata	-	0.78	3.13	3.13	3.13	3.13

-: Bacterial growth was not inhibitied at a maximum concentration of 12.50 mg/ml. *E. coli* WT: *E. coli* DHB4 strain. *E. coli ΔoxyR*: *E. coli* DHB4 strain with *oxyR* gene deletion.

Supplementary Table S2. MBC value of magnolol and honokiol against bacteria (μg/ml)

Bacterial type	Bacteria	Magnolol	Honokiol
Gram-negative bacteria	*E. coli*	>332	>332
	P. aeruginosa	>332	>332
Gram-positive bacteria	*S. aureus ATCC29213*	8	8
	S. aureus MRSA USA300 JE2	10	6
	S. epidermidis	8	8
	B. cereus	8	8
	B. subtilis	8	8

The diluted bacteria were cultured with magnolol or honokiol in a 96-well plate at 37˚C for 24 h. The bacteria include Gram-negative bacteria (*E. coli, P. aeruginosa*) and Gram-positive bacteria (*S. aureus, S. epidermidis, B. subtilis, B. cereus*) which were cultured to an OD600 ~0.4 and diluted 1,000 times. Whereafter, the MBC values were determined in triplicates using LB-agar medium plate.
